# 

**Published:** 1857-08

**Authors:** 


### CONTENTS.

### ORIGINAL COMMUNICATIONS.

PAGE

ARTICLE I.—A Case of Hypertrophy of the Spleen, and Post Mortem Examination. By WM. H. PHILPOT, M. D., of Redbone, Ga.,.................... 705
ARTICLE II.—Asthma—Its Pathology, Treatment, &c. By N. F. POWERS, M. D., Thompson, Ga.,.................... 708
ARTICLE III.—Convulsions in Children. By J. J. SCOTT, M. D., of Oxford, Mississippi,.................... 724

### SELECTIONS.

Sudden deaths in Puerperal state from Adynamic Lesions of the Nervous centers, etc.,.................... 727
The Family Physician,.................... 730
Criminal Abortion,.................... 733
Embalmment. Burial.................... 740

### EDITORIAL AND MISCELLANEOUS.

Atlanta Medical College,.................... 756
Society of Alumni of the Atlanta Medical College,.................... 757
The American Medical Association and Medical Education,.................... 757
Criminal Abortion or Fœticide,.................... 760
A Batch of Interesting questions,.................... 760
Preparatory Medical School,.................... 761
The Editor of the American Medical Gazette,.................... 761
To our Patrons,.................... 762
Commencement and Valedictory,.................... 762
University of Louisville—Medical Department,.................... 762
Jefferson Medical College,.................... 762
Dr. Fell's Cure for Cancer,.................... 763
National Hotel Disease,.................... 763
Iodine in Neuralgia.—Dr. James McClintock.—Veratrum Viride.—Excito-Secretory System of Nerves.—New Cure for Intermittent Fever,.................... 764
Treatment of Whooping Cough by Enemeta of Assafœtida.—Gelseminum Sempervirens.—Vesico Vaginal Fistula.—Amputation of the Cervix Uteri with the Ecraseur,.................... 765
Belladonna in Incontinence of Urine,.................... 766
Meteorological Observations for July, 1857,.................... 766
Veratrum Viride in Dysmenorrhœa,.................... 767
Subscriptions Received,.................... 767

